# EmojiGrid: A 2D Pictorial Scale for the Assessment of Food Elicited Emotions

**DOI:** 10.3389/fpsyg.2018.02396

**Published:** 2018-11-28

**Authors:** Alexander Toet, Daisuke Kaneko, Shota Ushiama, Sofie Hoving, Inge de Kruijf, Anne-Marie Brouwer, Victor Kallen, Jan B. F. van Erp

**Affiliations:** ^1^Human Factors, Netherlands Organisation for Applied Scientific Research (TNO), Soesterberg, Netherlands; ^2^Kikkoman Europe R&D Laboratory B.V., Wageningen, Netherlands; ^3^Research and Development Department, Kikkoman Corporation, Noda, Japan; ^4^Department of Microbiology and Systems Biology, Netherlands Organisation for Applied Scientific Research (TNO), Zeist, Netherlands; ^5^Research Group Human Media Interaction, University of Twente, Enschede, Netherlands

**Keywords:** affect grid, visual analog scales, EmojiGrid, emoji, emotion measurement

## Abstract

Research on food experience is typically challenged by the way questions are worded. We therefore developed the EmojiGrid: a graphical (language-independent) intuitive self-report tool to measure food-related valence and arousal. In a first experiment participants rated the valence and the arousing quality of 60 food images, using either the EmojiGrid or two independent visual analog scales (VAS). The valence ratings obtained with both tools strongly agree. However, the arousal ratings only agree for pleasant food items, but not for unpleasant ones. Furthermore, the results obtained with the EmojiGrid show the typical universal U-shaped relation between the mean valence and arousal that is commonly observed for a wide range of (visual, auditory, tactile, olfactory) affective stimuli, while the VAS tool yields a positive linear association between valence and arousal. We hypothesized that this disagreement reflects a lack of proper understanding of the arousal concept in the VAS condition. In a second experiment we attempted to clarify the arousal concept by asking participants to rate the valence and intensity of the taste associated with the perceived food items. After this adjustment the VAS and EmojiGrid yielded similar valence and arousal ratings (both showing the universal U-shaped relation between the valence and arousal). A comparison with the results from the first experiment showed that VAS arousal ratings strongly depended on the actual wording used, while EmojiGrid ratings were not affected by the framing of the associated question. This suggests that the EmojiGrid is largely self-explaining and intuitive. To test this hypothesis, we performed a third experiment in which participants rated food images using the EmojiGrid without an associated question, and we compared the results to those of the first two experiments. The EmojiGrid ratings obtained in all three experiments closely agree. We conclude that the EmojiGrid appears to be a valid and intuitive affective self-report tool that does not rely on written instructions and that can efficiently be used to measure food-related emotions.

## Introduction

### Background

Besides the sensory characteristics of food, food-evoked emotion is a crucial factor in predicting consumer’s food preference and therefore in developing new products (Dalenberg et al., 2014; Gutjar et al., 2015). Hedonic ratings alone do not predict food choice behavior accurately (Wichchukit and O’Mahony, 2010, 2011). Dalenberg et al. (2014) showed that consumers’ emotions add predictive power to a food choice (predicting) model based on hedonic scales, while Gutjar et al. (2015) found that self-reported food-evoked emotions can predict individual’s food choice more accurately than hedonic scores. These studies suggest that assessing emotional responses to foods may reveal product attributes which can be a valuable source of information for product development and marketing that goes beyond traditional sensory and acceptability measurements (Thomson et al., 2010; Jaeger et al., 2018b). Therefore, it seems important to obtain valid and reliable measurements of food-evoked emotions. According to the circumplex model of affect (Russell, 1980), emotions are characterized by both their valence (pleasantness; the degree of positive or negative affective response to a stimulus) and arousal (the intensity of the affective response to a stimulus; the degree of activation or deactivation). Since hedonic ratings alone do not predict food choice behavior accurately (Wichchukit and O’Mahony, 2010, 2011), it appears that both valence and intensity play a distinct and critical role in eating-related behavior (Woodward et al., 2017).

Human affective response to food can be assessed objectively by measuring the user’s behavioral (e.g., amount consumed, facial expressions) and physiological (e.g., electrodermal activity, heart rate) signals and subjectively using affective self-report tools (e.g., questionnaires, affective lexicons, graphical scales; for a recent review of all different assessment methods see Kaneko et al., 2018).

Affective self-report questionnaires are the most widely used tools since they are extensively validated and easy to apply. These tools can be divided into two main groups: tools that represent emotions verbally (e.g., through names like “*fear*,” adjectives like “*afraid*” or even full sentences: King and Meiselman, 2010; Spinelli et al., 2014; Nestrud et al., 2016) and tools that represent emotions graphically (e.g., through smiling or frowning faces: Bradley and Lang, 1994; Vastenburg et al., 2011; Laurans and Desmet, 2012; Broekens and Brinkman, 2013; Huisman et al., 2013; Obaid et al., 2015).

Verbal tools enable users to report their current affective state by selecting or rating words that best express their feelings. They are the most commonly used techniques to measure emotional responses to food, due to their ease of application, cost-effectiveness, and discriminative power (Churchill and Behan, 2010; Dorado et al., 2016). However, they have several shortcomings: (1) affect and emotions (especially mixed or complex ones) are difficult to verbalize and the labels used to describe them are inherently ambiguous (Scherer, 2005; Köster and Mojet, 2015) and (2) the “affective” or “emotional” lexicon varies across cultures and languages, particularly when it comes to foods (e.g., Curia et al., 2001; Gutjar et al., 2015; van Zyl and Meiselman, 2015). Also, verbal tools are demanding for the user since they require cognitive effort (interpretation) and a significant amount of time to fill them out. This disadvantage increases when they need to be repeatedly applied in the course of an experiment.

Graphical tools allow users to report their feelings efficiently and intuitively by indicating or rating the (part of the) figure that best represents their current affective state. Graphical self-report instruments are appealing for the measurement of affective experiences since they do not require the users to verbalize their emotions. Instead, they rely on the human ability to intuitively and reliably attribute emotional meaning to (simple) graphical elements (Aronoff et al., 1988; Windhager et al., 2008; Larson et al., 2012; Watson et al., 2012), in particular those linked to facial expressions (Tipples et al., 2002; Lundqvist et al., 2004; Weymar et al., 2011). It has therefore been suggested to replace the subjective linguistic increments on rating scales by iconic facial expressions (Kaye et al., 2017). Since graphical self-report tools do not rely on verbal descriptions of emotions, they may also be useful for cross-cultural studies since they eliminate the need for translation and the problems associated therewith (e.g., Curia et al., 2001; van Zyl and Meiselman, 2015). Also, they may be more effective to measure and express mixed (complex) emotions that are hard to verbalize (Elder, 2018). Hybrid tools that combine graphical elements with verbal labels to clarify their meaning (e.g., Cowie et al., 2000) may be useful for populations with inherent reading problems (e.g., dyslexia).

In the next section, we first give a brief overview of existing affect self-report measurement tools, focusing in particular on pictorial scales, and we discuss their limitations as tools to measure food-related emotional experiences.

### Related Work

#### Affective Self-Report Through Cartoon Characters

The Affect Grid (Russell et al., 1989) is a two-dimensional labeled visual scale to assess affect along the principal dimensions valence and arousal, based on Russell (1980)’s circumplex model of affect. The horizontal valence scale ranges from “*unpleasant*” (low negative valence) to “*pleasant*” (high positive valence). The vertical arousal scale ranges from “*sleepiness*” (low intensity – no arousal) to “*high arousal*” (high intensity). Four additional labels (“*stress*,” “*excitement*,” “*depression*,” and “*relaxation*”) clarify the meaning of the extreme emotions represented by the corners of the grid. Users mark the location on the grid that best corresponds to their affect state after perceiving a given stimulus. Hybrid abstract and pictorial versions of the Affect Grid have been created by labeling its axes either with icons of faces showing different emotional expressions (Schubert, 1999) or with abstract cartoon characters (Swindells et al., 2006; Cai and Lin, 2011). Although the Affect Grid has been applied to measure food elicited emotions (Einöther et al., 2015; den Uijl et al., 2016b), none of these tools has been specifically designed to assess food-related emotions.

Other affective self-report tools use cartoon characters that express specific emotions through facial and bodily expressions. The rationale for their use is twofold. First, people can accurately identify discrete emotions from bodily signals such as facial expressions (Ekman, 1994) and body language (Wallbott, 1998) across cultures (Ekman and Friesen, 1971). Second, visually expressed emotions are hypothesized to more closely resemble intuitively experienced emotions (Dalenberg et al., 2014). Evidence for this hypothesis stems from EEG experiments showing that emotion processing is faster for facial expressions than for emotional words (Schacht and Sommer, 2009; Frühholz et al., 2011; Rellecke et al., 2011). Although none of the currently available cartoon-based self-assessment tools have been designed to measure food-related emotions, we will first give a brief overview of the existing methods since they are closely related to the new tool that we will present later in Section 2.

The Self-Assessment Manikin (SAM; Bradley and Lang, 1994) is a pictorial assessment technique that enables users to report their momentary feelings of valence, arousal, and dominance by selecting for each factor from a set of humanoid figures showing different intensities the one that best expresses their own feeling. Muñoz et al. (2010) introduced an additional SAM scale to measure food-related craving (the desire to consume; see also Miccoli et al., 2014). Although the SAM is widely used and extensively validated, it is generally acknowledged that it has several serious drawbacks. First, users often misunderstand the depicted emotions. Especially children have difficulties understanding the SAM (Yusoff et al., 2013; Hayashi et al., 2016). While the valence dimension of the SAM is quite intuitive (depicted as the figure’s facial expression going from a frown to a smile), the dominance dimension (depicted as the size of the figure) is much harder to explain, and the arousal dimension (depicted as an “explosion” in the stomach area) is often misinterpreted (Broekens and Brinkman, 2013; Betella and Verschure, 2016; Chen et al., 2018). Second, the method still requires a successive assessment of the stimulus on multiple dimensions separately.

Product Emotion Measurement Instrument (PrEmo) is a non-verbal cross-cultural validated self-report instrument to measure 14 distinct emotions visualized by an animated cartoon character (Desmet et al., 2000; Laurans and Desmet, 2012). Users rate to what extent the animated figures express their feelings elicited by a stimulus, using a five-point scale. Although PrEmo has been applied to measure food elicited emotions (Dalenberg et al., 2014; Gutjar et al., 2015; den Uijl et al., 2016b; He et al., 2016a,b), it was not designed for this purpose and most of the displayed emotions (e.g., *pride, hope, fascination, shame, fear, sadness*) therefore have no evident relation to food experiences. Similar cartoon-based self-report tools representing a limited set of emotions are the Pictorial Mood Reporting Instrument (PMRI; Vastenburg et al., 2011), the pictorial ERF (Emotion Rating Figurines; Obaid et al., 2015), the LEMtool (Layered Emotion Measurement tool; Huisman and van Hout, 2008; Huisman et al., 2013), and Pick-A-Mood (Desmet et al., 2016). The Affective Slider is a digital scale composed of two vertically aligned sliders labeled with stylized facial expressions that represent pleasure and arousal (Betella and Verschure, 2016). Unlike the previous methods, the AffectButton (Broekens and Brinkman, 2013) and EMuJoy (Emotion measurement with Music by using a Joystick; Nagel et al., 2007) allow users to continuously adjust the emotional expression of a cartoon character (by moving a mouse controlled cursor). However, these tools require the user to successively explore the entire affective space to find the desired expression each time a response is given, unlike the other graphical tools that provide an instantaneous overview of the affective input space.

#### Affective Self-Report Through Emoji

Emoji are pictographs or ideograms representing emotions, concepts, and ideas. They are widely used in electronic messages and Web pages to supplement or substitute written text (Danesi, 2016). Facial emoji are typically used to change or accentuate the tone or meaning of a message. They can support users to express and transmit their intention more clearly and explicitly in computer-mediated communication (dos Reis et al., 2018). Emoji span a broad range of emotions, varying in valence (e.g., smiling face vs. angry face) and arousal (e.g., sleepy face and face with stuck-out tongue and winking eye). Although some facial emoji can be poly-interpretable (Miller et al., 2016; Tigwell and Flatla, 2016) it has been found that emoji with similar facial expressions are typically attributed similar meanings (Moore et al., 2013; Jaeger and Ares, 2017) that are also to a large extent language independent (Kralj Novak et al., 2015). Emoji can elicit the same range of emotional responses as photographs of human faces (Moore et al., 2013). In contrast to photographs of human faces, emoji are not associated with overgeneralization (the misattribution of emotions and traits to neutral human faces that merely bear a subtle structural resemblance to emotional expressions; Said et al., 2009), or racial, cultural, and sexual biases.

For a study on children’s sensitivity to mood in music, Giomo (1993) developed a non-verbal response instrument using schematic faces arranged in a semantic differential format along three lines corresponding to each of the three musical mood dimensions defined by Wedin (1972). By marking the most appropriate facial expression children used the tool to report their perceived mood in musical pieces.

Schubert (1999) developed the interactive Two-Dimensional Emotion-Space (2DES) graphic response tool to enable continuous measurement of perceived emotions in music. The 2DES tool consists of a square Affect Grid (with valence along the horizontal and arousal along the vertical axis) with schematic faces (showing only eyes and a mouth) arranged at the corners and the midpoints of the four sides of the grid. No further labels are provided. The human–computer interface records cursor movements within the square. The schematic faces represent the arousal dimension by the size of the mouth and the eye opening, while the valence dimension is represented by the concavity of the mouth. These features are based on the literature on facial expression (Ekman et al., 1971). An extensive evaluation study showed that the instrument was intuitive to use and had a significant reliability and validity (Schubert, 1999). The author suggested that the tool could be applied to measure emotion felt in response to a stimulus rather than emotion expressed by the stimulus (Schubert, 1999).

Russkman (Russell and Ekman; Sánchez et al., 2006) is an interactive graphic response tool consisting of a set of emoji expressing 28 affective states on three levels of intensity. Russkman is based on Russell (1980)’s circumplex model of affect and Ekman’s facial Action Coding System (FACS; Ekman and Rosenberg, 2004) and was developed to convey mood and emotion in instant messaging. The user can select a specific emotion by moving a cursor on top of one of the four icons representing the quadrants of an Affect Grid, which then expands making all icons in this quadrant available for selection.

To make the SAM more accessible to children, Hayashi et al. (2016) replaced the cartoon characters with emoji. Their five-point “emoti-SAM” was quickly grasped by children and effectively used as both an online and a paper version.

Swaney-Stueve et al. (2018) developed a seven-point bipolar valence scale labeled with emoji. They compared this scale to a nine-point verbal liking scale in an online experiment in which children reported their affective responses to different pizza flavors and situations. Both scales yielded similar responses distributions with a strong positive linear correlation (*R*^2^ > 0.99 for both pizza flavors and situations). They concluded that further research was needed to extend their unidimensional emoji scale into a two-dimensional one that also measures arousal.

Emoji-based rating tools are increasingly becoming popular tools as self-report instruments (Kaye et al., 2017) to measure for instance user and consumer experience (e.g., www.emojiscore.com). For instance, Moore et al. (2013) developed a nine-point emoji scale to measure users’ affective responses to an online training simulation, and Alismail and Zhang (2018) used a five-point emoji scale to assess user experience with electronic questionnaires. While emoji typically express different degrees of valence and arousal (Moore et al., 2013), previous studies only validated (Aluja et al., 2018) and used (Moore et al., 2013; Alismail and Zhang, 2018) the valence dimension.

While people do not easily name food-related emotions, they appear to use emoji in a spontaneous and intuitive way to communicate food-related emotional experiences (Vidal et al., 2016). Previous studies found that emoji can serve as a direct self-report tool for measuring food-related affective feelings (Vidal et al., 2016; Ares and Jaeger, 2017; Gallo et al., 2017; Jaeger et al., 2017b, 2018a; Schouteten et al., 2018). However, these previous studies used subsets of the most popular and currently available emoji, most of which show facial expressions that have no clear relation to food experiences. Also, the size of these sets (33 emoji: Ares and Jaeger, 2017; Jaeger et al., 2017b, 2018a; Schouteten et al., 2018; 25–39 emoji: Jaeger et al., 2017a; and 50 emoji: Gallo et al., 2017) is rather overwhelming and comparable to the large number of words typically used in emotional lexicons to measure emotional associations to food and beverages (e.g., King and Meiselman, 2010; Spinelli et al., 2014; Nestrud et al., 2016). These large set sizes make emoji-based rating or selection procedures quite inefficient. Sets of emoji were used in both check-all-that-apply (CATA) (Ares and Jaeger, 2017; Jaeger et al., 2017a,b; Schouteten et al., 2018) and rate-all-that-apply (RATA; Ares and Jaeger, 2017) questionnaires. In general, these studies found that emoji are capable to discriminate well between hedonically diverse stimuli, while the reproducibility of the emotional profiles was quite high (Jaeger et al., 2017b). Compared with other non-verbal methods that use cartoon figures to represent different emotions (e.g., Desmet et al., 2012; Laurans and Desmet, 2012; Huisman et al., 2013), emoji characters appear to have the advantage of being more familiar to users. It seems that users easily connect emoji to food-elicited emotions, even without any explicit reference to feelings in the wording of the associated question (Ares and Jaeger, 2017). Given that emotions in facial expressions, gestures, and body postures are similarly perceived across different cultures (Ekman and Friesen, 1971; Ekman, 1994), cross-cultural differences in the interpretation of emoji could also be smaller than the influences of culture and language on verbal affective self-report tasks (Torrico et al., 2018). Also, emoji provide a visual display of emotion, making them also beneficial for use with children who may not have the vocabulary to convey all their emotions (Gallo et al., 2017; Schouteten et al., 2018).

For repeated or routine testing in applied settings, selecting emoji from a long list of possible candidates may be a task that is too demanding, and shorter tests are therefore required. The emoji used to measure food-related emotions in previous studies (Ares and Jaeger, 2017; Gallo et al., 2017; Jaeger et al., 2017a,b; Schouteten et al., 2018) were not specifically developed for this purpose but were merely selected as the most appropriate ones from the general set of available emoji. As a result, several emoji were obviously out of context and had no relevance for the description food-related affective associations (Jaeger et al., 2017b). Also, the most frequently used emoji are primarily associated with positive emotional experiences reflecting the dominance of positive emotions in food consumption (hedonic asymmetry; Desmet and Schifferstein, 2008). Hence, there is a need for a set of emoji that (1) specifically relate to food experience and (2) that span the entire hedonic continuum from negative to positive emotions.

### Current Study

In the previous section, we identified a need for an efficient food-specific graphical (language independent) affective self-report method that produces reliable and valid data. We also identified emoji as a promising graphical avenue.

In the rest of this paper, we first introduce and validate the EmojiGrid, which is a new efficient graphical self-report tool that can measure food-related affective states along the dimensions of valence and arousal.

Then we will present the results of two comparative evaluation studies in which participants rated valence and arousal of images of food either with the new EmojiGrid scale or with conventional visual analog scales (VAS). Previous research has shown that viewing pictures of food not only activates the visual cortex, but also brain areas that code how food actually tastes (the insula/operculum) and the reward values of tasting it (the orbitofrontal cortex; Simmons et al., 2005). Food images are therefore typically considered a viable surrogate for the real thing (e.g., Foroni et al., 2013; Blechert et al., 2014; Miccoli et al., 2014, 2016). Finally, we will present some conclusions and suggestions for future research.

## Emojigrid: Design and Validation

**FIGURE 1:**
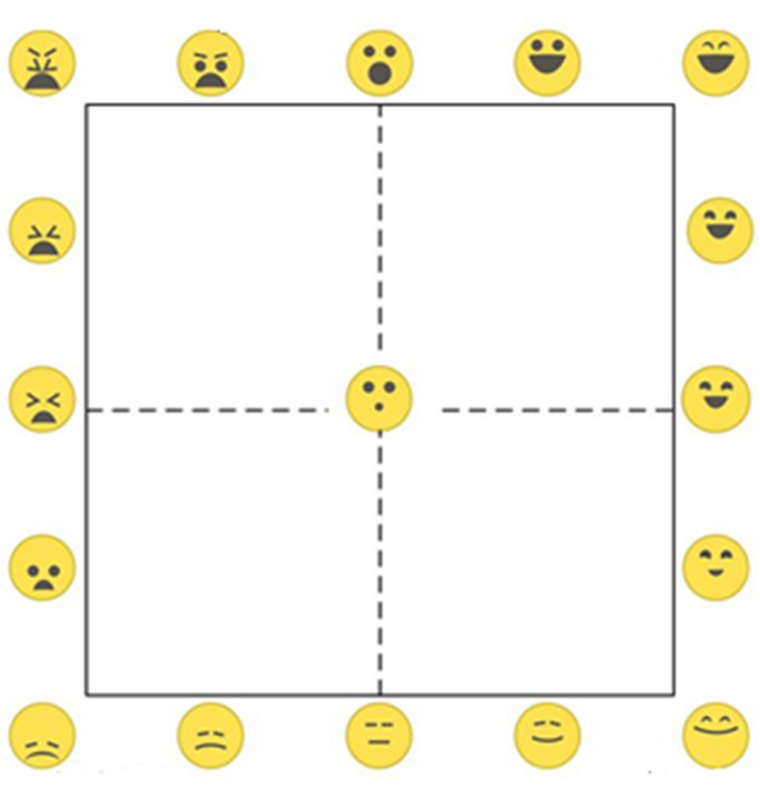
The EmojiGrid: an emoji-labeled Affect Grid for the measurement of food-related affective associations.

Here we propose the EmojiGrid (Figure 1) as a new tool to assess food-related affective associations.

### Design

The EmojiGrid is a Cartesian grid similar to the Affect Grid (Russell et al., 1989), but the verbal labels are replaced with emoji showing food-related facial expressions. Also, additional emoji are inserted between the midpoints and the endpoints of each axis (resulting in five emoji on each side of the grid), and one emoji is placed in the center of the grid, resulting in a total of 17 emoji on the grid. A central neutral expression serves as a baseline or anchor point. The facial expressions vary from disliking (unpleasant) via neutral to liking (pleasant) along the horizontal (valence) axis, and gradually increase in intensity along the vertical (arousal) axis. The facial expressions are defined by the eyebrows, eyes, and mouth configuration of the face, and are inspired by the FACS (Ekman and Friesen, 2003). The arousal dimension is represented by the opening of the mouth and the shape of the eyes, while the valence dimension is represented by the concavity of the mouth, the orientation, and curvature of the eyebrows, and the vertical position of these features in the face area (representing a slightly downward looking face for lower arousal values and a slightly upward looking face for higher valence values). These facial features represent a minimal set needed to express the range of emotions over the Affect Grid. To avoid potential biases in ratings due to the emotional connotation of colors (Clarke and Costall, 2008; Suk and Irtel, 2010), we adopted a monochromatic (yellow) color scheme in the design of the EmojiGrid. Users place a check mark at the location in the grid that corresponds to the emoji (facial expression) that best represents their affective state (feelings) after perceiving a certain food or beverage.

Previous studies using emoji to measure food-evoked emotions typically started with a large set of currently available (extremely heterogeneous) emoji and merely selected those emoji that could somehow be related to food (e.g., Ares and Jaeger, 2017; Gallo et al., 2017; Jaeger et al., 2017a,b, 2018a; Schouteten et al., 2018). This approach typically results in a limited set of emoji with widely different (and not systematically varying) characteristics, that also does not cover the full valence–arousal space. The emoji used to label the EmojiGrid were designed to represent facial expressions corresponding to the emotions represented by the grid points along the outer edges of the Affect Grid that represents the general affective dimensions of valence and arousal. Hence, the iconic facial expressions of the emoji represent emotions that can be induced by any stimulus or event, including food. Thus, the stimuli were not specifically designed to reflect only food-induced emotions. The systematic variation in the shape and size of the facial characteristics (eyebrows, eyes, and mouth) of the emoji enables users to interpolate facial expressions between the label icons on the edges of the grid.

### Validation

We performed three validation studies to assess whether the emoji had indeed the intended and intuitive order across the valence–arousal space. The tasks involved an integral interpretation of the shape and size of the mouth and eyes and the position and shape of the eyebrows. While, as noted before, the stimuli were designed to represent general emotions and not merely reflect food-induced emotions, the facial expression was such that all of them could be related to food-induced emotions.

#### Affective Assessment of Individual Emoji

To validate the EmojiGrid, a convenience sample of 28 Dutch students (18 females, 10 males), aged between 18 and 24 years, rated each individual emoji label on valence and arousal, using five-point SAM scales. The emoji were presented in random order.

Pearson’s correlation between the SAM valence and arousal ratings and the scale values corresponding to the position of the emoji on the EmojiGrid (i.e., the label indices) was, respectively, *r*(15) = 0.96 and 0.92, *p* < 0.01, indicating close agreement between both scales. This result agrees with that of Swaney-Stueve et al. (2018), who found that a seven-point valence scale labeled with emoji and a verbal liking scale yielded similar responses distributions with a strong positive linear correlation (*R*^2^ > 0.99 for both pizza flavors and situations).

In this experiment the emoji were individually presented in random order. In the actual EmojiGrid they are arranged along the edges in order or increasing valence and arousal. We hypothesize that this linear spatial arrangement along the edges will serve to provide a correct impression of the corresponding gradual variation in facial expression (representing either valence or arousal).

#### Linear Ordering Emoji of Similar Valence or Arousal

**FIGURE 2:**
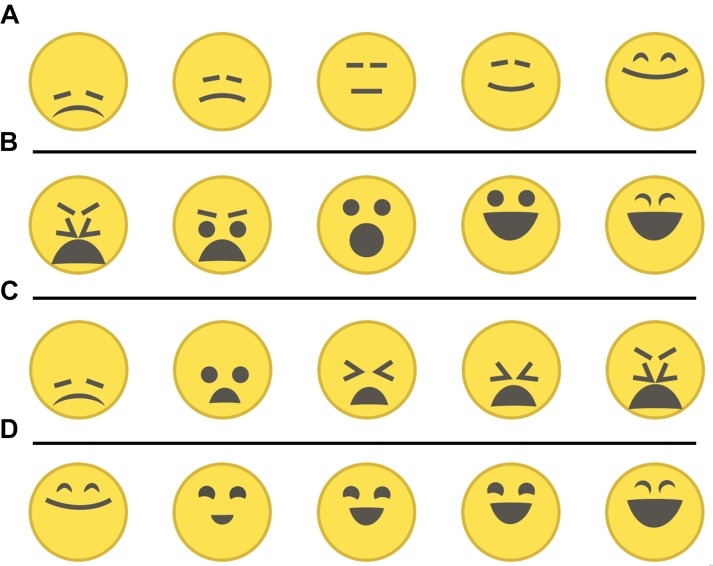
The four sets of five emoji each used in the ranking experiment, representing the emoji along **(A)** the bottom edge (lowest arousal), **(B)** top-edge (highest arousal), **(C)** left edge (lowest valence), and **(D)** right edge (highest valence).

To test the hypothesis that a linear arrangement can enhance the perception of the logical order of the emoji labels on the edges of the EmojiGrid, a convenience sample of 10 Dutch students (five females, five males), aged between 19 and 25 years, ordered four sets of five emoji each. The four sets represented, respectively, the emoji labels on the bottom edge (low arousal; Figure 2A), top edge (high arousal; Figure 2B), left edge (low valence; Figure 2C), and right edge (high valence; Figure 2). The participants were asked to order the two sets of emoji with similar (low or high) arousal (Figures 2A,B) along a line segment in order of increasing valence and the two sets of emoji with similar (low or high) valence (Figures 2C,D) in order or increasing arousal. The stimuli were presented in PowerPoint slides. The emoji were initially randomly ordered on top of the screen, and the participants could drag them to the lines segment using their mouse.

The two sets of emoji with similar arousal values (Figures 2A,B) were both correctly ranked in order of increasing valence by all participants. For the two sets of emoji with similar valence values (Figures 2C,D), only the first two emoji (i.e., the ones representing the lowest level of arousal) were ranked in reverse order by 3 (out of 10) participants. This reversal of emoji representing low arousal may be resolved when a valence context is provided (i.e., when the arousal axes are flanked by their corresponding valence axes, as in the EmojiGrid).

#### Circular Ordering of Emoji

To test the hypothesis that people are able to correctly order the labels of the EmojiGrid when their full valence–arousal context is provided, we asked participants to arrange the 16 emoji that are used as labels on the edges of the EmojiGrid along a circle (the topological equivalent of the boundary of the EmojiGrid) in a “logical order.” The stimuli were presented in PowerPoint slides. The 16 emoji were initially displayed in a random order on the upper part of the screen, and a large circle was shown on the lower part of the screen. The participants could use their mouse to place the emoji anywhere along the circle using their mouse. A convenience sample of 10 Dutch students (five females, five males), aged between 21 and 25, ordered all 16 emoji. Most participants correctly ordered the emoji (i.e., in the same order as they have along the EmojGrid). Two participants reversed the order of the neutral emoji with the highest valence (3rd emoji in Figure 2B) and the emoji with the lowest valence and second lowest arousal (2nd emoji in Figure 2C).

### Discussion

Overall, this validation study shows that the individual emoji reliably convey the intended degrees of valence and arousal, and that their arrangement along the boundaries of the EmojiGrid appears intuitive.

## General Methods

### Measures

In this study participants rated the valence and arousal of food images using either the new EmojiGrid or labeled VAS. The participants responded by positioning a cursor on the appropriate location of the respective scales and clicking with the mouse button. The two dimensions of the EmojiGrid and the two VAS (corresponding to the affective dimensions of valence and arousal) were all converted to a range from 0 to 100 points.

### Participants

Participants with a Dutch nationality were recruited through postings on social media and direct emailing. The experimental protocol was reviewed and approved by the TNO Ethics Committee (Ethical Application Ref: 2017-011) and was in accordance with the Helsinki Declaration of 1975, as revised in 2013 (World Medical Association, 2013). Participation was voluntary. All participants gave their web-based informed consent instead of written consent. After completing the study the participants were offered to participate in a raﬄe for vouchers for an online shopping site, with a value of 10 Euros each.

### Stimuli

**FIGURE 3:**
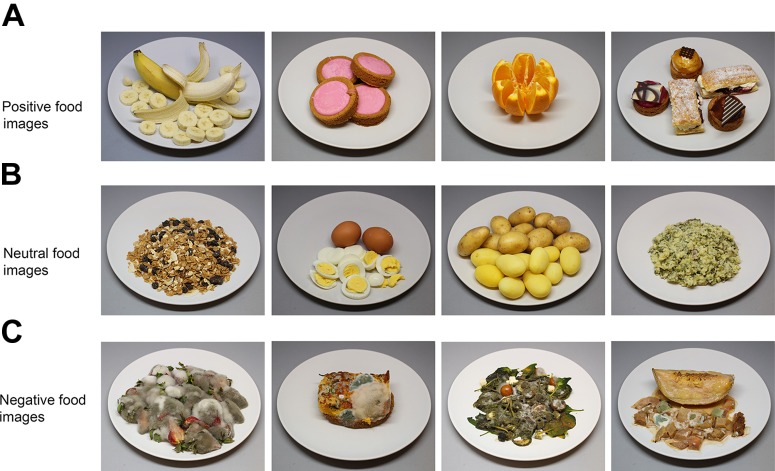
Stimulus examples. **(A)** Positive food images (banana, cookies, orange, sweets), **(B)** neutral food images (cereals, boiled eggs, boiled potatoes, hotchpot), and **(C)** negative images of rotten food (strawberries, omelet on bread, Greek salad, melon).

**FIGURE 4:**
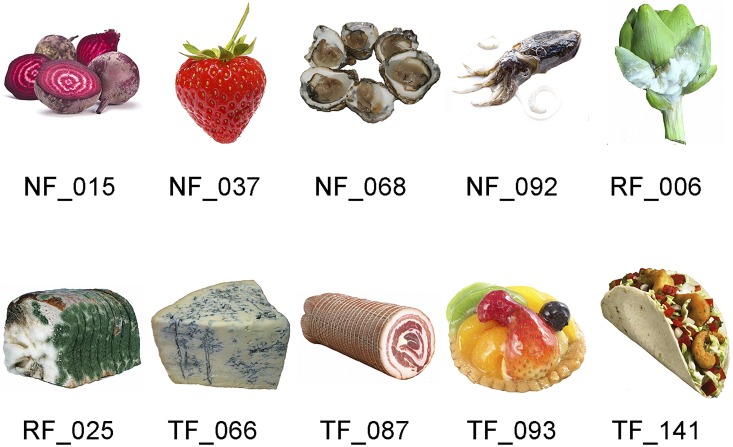
The 10 images from the FRIDa database (images reproduced with the permission of the copyright holder: Foroni et al., 2013). The labels are the original identifiers of the images in the FRIDa database (NF, natural food; RF, rotten food; TF, transformed food). This set consists of two highly negative (RF) and three highly positive (NF_037, TF_093, and TF_141) images. The remaining five images are distributed over the neutral zone of the valence–arousal continuum.

The stimulus set consisted of 60 different food images: 50 images were specifically registered for this study according a standard protocol [see Charbonnier et al. (2016); for some examples see Figure 3] and 10 additional images were taken from the FoodCast research image (FRIDa) database (Foroni et al., 2013; Figure 4). The 50 images that were registered for this study (Figure 3) have a resolution of 1037 × 691 pixels and represent natural food (e.g., strawberry, salad), rotten or molded food (e.g., rotten banana, molded salad), raw food (e.g., raw chicken, raw potatoes), processed food (e.g., cakes, fried fish), unfamiliar food (e.g., locusts), and contaminated food (e.g., hotchpot with fake turd). The set of food items was selected such that their perceived valence is likely to be distributed along the entire valence scale (ranging from very low valence for rotten, molded, or contaminated food, via neutral for raw onions, boiled eggs, or potatoes, to very high valence for fresh fruit, chocolates, and pastries). The 10 additional food images from the FRIDa database (Figure 4) have a resolution of 530 × 530 pixels and were selected such that their associated valence scores (as reported in their accompanying data file, see Foroni et al., 2013) were approximately evenly distributed over the full range of the valence space covered by this dataset (i.e., ranging from minimal to maximal valence and in between). Five of the images had positive valence (squid/NF_093, ham/TF-087, tacos/TF_141, strawberry/NF_037, and tart/TF_093), the other five had negative valence (molded bread/RF_025, sprout/RF_006, Oyster/NF_068, beetroots/NF_015, and blue cheese/TF_066). The validated FRIDa images were included as anchor points for verification purposes: in Experiment 1 of the current study, the VAS valence and arousal ratings for these images were obtained following the same procedure as used in the study by Foroni et al. (2013).

### Procedure

Participants took part in an anonymous online survey. Although Internet surveys typically provide less control over the experimental conditions, they typically yield similar results as lab studies (e.g., Gosling et al., 2004; Woods et al., 2015; Majima et al., 2017) while they limiting several disadvantages associated with central location studies.

The experiment was programmed in the Java script language, and the survey itself was hosted on a web server. The time stamps of the different events (onset stimulus presentation, response clicks) and the display size and operating system of the participants were logged. This enabled us to check that participants did indeed view the stimuli on larger displays and not on mobile devices with low resolution screens. The resolution of the devices used by the participants in this study varied between 1280 × 720 and 3440 × 1440 (the average resolution was 1538 × 904 pixels across participants, with standard deviations of 330 × 165 pixels). We could not verify if the browser window was indeed maximized.

The survey commenced by presenting general information about the experiment and thanking participants for their interest. Also, the participants were asked to put their web browser in full-screen mode to maximize the questionnaire resolution and avoid external distractions such as software running in the background. Then the participants were informed that they would see 60 different food images during the experiment and they were instructed to rate their first impression of each image without worrying about calories. It was emphasized that there were no correct or incorrect answers and that it was important to respond seriously. Subsequently, participants electronically signed an informed consent by clicking “*I agree to participate in this study*,” affirming that they were at least 18 years old and voluntarily participated in the study. The survey then continued with an assessment of the demographics and the current physical (degree of hunger and thirst, fullness) state of the participants.

Next, the participants were shown either the VAS or the EmojiGrid response tool (depending on the experimental condition to which they were randomly assigned) together with an explanation about how they should use the tool to report their (valence and arousal) ratings for each image.

**FIGURE 5:**
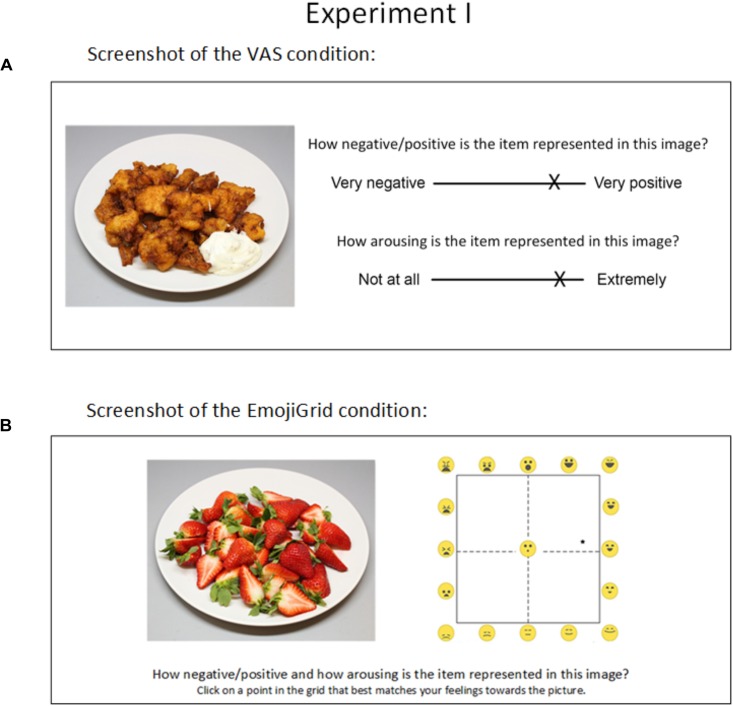
Screenshot of the VAS **(A)** and EmojiGrid **(B)** rating conditions in Experiment I.

On each trial the screen displayed the image of a food item on the left side of the screen and the response tool (depending on the experimental condition either a VAS or the EmojiGrid) on the right side of the screen (see e.g., Figure 5). After giving a response by clicking on the (VAS or EmojiGrid) response tool, the next food image appeared, and the response tool was reset (the check mark was removed from the response tool). The presentation duration of each stimulus was not restricted.

The participants first performed two practice trials to familiarize them with the use of the response tool. Immediately after these practice trials, the actual experiment started. The 60 different food images were randomly presented over the course of the experiment. The entire experiment was self-paced and lasted typically about 15 min (the mean duration was 14.59 ± 2.32 min).

To assess the seriousness of the participation we included a validated seriousness check (Aust et al., 2013). After completing the experiment, participants were asked whether they had answered in a serious manner. The wording of the question was as follows: “*It would be very helpful if you could tell us at this point whether you have taken part seriously, so that we can use your answers for our scientific analysis, or whether you were just clicking through to take a look at the experiment*?” Participants were able to choose one of two answers: “*I have taken part seriously*” or “*I have just clicked through, please throw my data away*.” To further motivate the participants, we also included a seriousness question at the start of the experiment: “*It’s important for us that you are motivated and answer all questions seriously.*” All participants indicated that they took the experiment seriously.

### Data Analysis

Matlab 2018a^1^ was used to analyze the data and plot the results. For each image and for both self-assessment tools (EmojiGrid and VAS) we computed the mean response across all participants. To get an impression of the agreement between the performance of both response tools we computed the linear correlation between the ratings obtained with both methods for valence and arousal separately. For both tools, we investigated the relation between the valence and arousal ratings by computing least-squares fits of either linear or quadratic functions to the data points.

In this study we checked the occurrence of a random answering behavior by inspecting the consistency of ratings given for stimuli with the highest and lowest overall valence ratings (corresponding, respectively, to pleasant food items with an overall high positive mean valence rating and unpleasant ones with an overall low negative mean valence rating). We did not observe any outliers, in the sense that there were no participants that gave positive ratings to stimuli with overall mean low valence ratings or the other way around. This suggests that random answering behavior did not occur in this study.

## Experiment I: Food Valence and Arousal Measured With Emojigrid and VAS

This experiment was performed to compare the performance of the EmojiGrid with conventional VAS. Dutch participants rated the valence and the arousing quality of 60 different food images, using either the EmojiGrid or two independent conventional VAS. The VAS procedure exactly replicated a procedure that was used previously in a similar study in the literature (Foroni et al., 2013) thus enabling to us compare the performance of both methods.

### Materials and Methods

#### VAS

In the VAS condition, the participants were asked to rate how each image made them feel by using two scales: one for valence and one for arousal (Figure 5A). The valence scale measured the perceived pleasantness of the displayed product. The question associated with this scale was: “*How negative/positive is the item represented in the image*?” and the extremes of the scale were labeled “*Very negative*” and “*Very positive*.” The arousal scale measured the excitement that was experienced while viewing the image. The question associated with this scale was: “*How arousing is the presented image*?” and the extremes of the scale were labeled “*Not at all*” and “*Extremely*.” This procedure exactly replicates the one used by Foroni et al. (2013) to assess valence and arousal for the images in the FRIDa database.

#### The EmojiGrid

In the EmojiGrid condition, participants rated their affective feelings toward each image on the dimensions of valence and arousal by responding to the question “*How negative/positive and how arousing is the item represented in the image*?” using the EmojiGrid, with the additional instruction “*Click on a point in the grid that best matches your feelings toward the picture*” (Figure 5B).

#### Participants

The total sample consisted of *N* = 136 participants, 66 males and 70 females, with a mean age of *M* = 39.21 (*SD* = 13.28). Participants were randomly assigned to one of the two experimental conditions.

The sample in the VAS condition consisted of *N* = 57 participants, 28 males and 29 females with a mean age of *M* = 42.28 (*SD* = 13.92).

The sample in the EmojiGrid condition consisted of *N* = 79 participants, 38 males and 41 females with a mean age of *M* = 37 (*SD* = 12.42).

### Results

#### EmojiGrid Versus VAS

**FIGURE 6:**
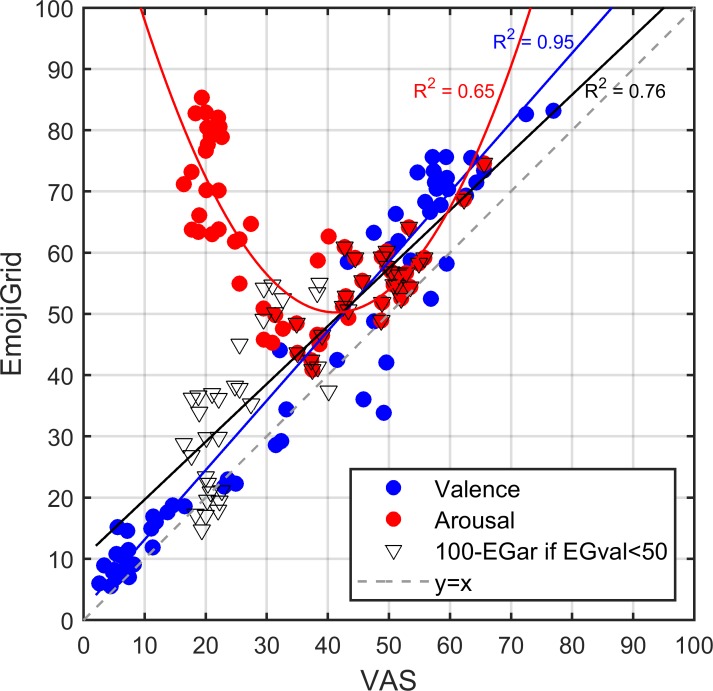
Relation between corresponding mean affective ratings obtained with the VAS and EmojiGrid in Experiment I. Blue dots: mean valence ratings. Red dots: mean arousal ratings. Black triangles: mean arousal ratings after inverting the EmojiGrid values for items that were rated as unpleasant (i.e., for which the corresponding valence was lower than 50). The blue and red lines represent linear fits to the valence and arousal ratings. The black line represents a linear fit to the partially inverted EmojiGrid scores. The broken gray line with slope 1 represents full agreement between both methods.

For each image and for both self-assessment tools (EmojiGrid and VAS) we computed the mean response across all participants. Figure 6 shows the relation between corresponding affective ratings obtained with both tools. This figure suggests an overall linear relation between the valence ratings obtained with both methods, while the relation between the arousal ratings appears to be U-shaped. To get a first impression of the agreement between the performance of both response tools we computed the linear (Pearson) correlation between the ratings obtained with both methods for valence and arousal separately. The valence ratings showed a strong overall positive association between both methods (*r* = 0.97, *p* < 0.001). A least-squares linear fit to the data points (blue line in Figure 6) confirmed this observation (adjusted *R*-squared of 0.92). The arousal ratings showed a moderate and negative association between both methods (*r* = -0.47, *p* < 0.001).

To further quantify the agreement between both methods we also computed intraclass correlation coefficient (ICC) estimates and their 95% confidence intervals, based on a mean-rating, absolute-agreement, two-way mixed-effects model (Table 1; Shrout and Fleiss, 1979; Koo and Li, 2016). The ICC for valence was 0.958 (with a 95% confidence interval ranging between 0.690 and 0.986) and the ICC for arousal was -0.491 (with a 95% confidence interval ranging between -0.930 and 0.308), indicating that the valence ratings obtained with both methods (EmojiGrid and VAS) are in excellent agreement, while there is no agreement between the arousal ratings.

**Table 1:** Intraclass correlation coefficients for the mean valence and arousal ratings obtained with the VAS and EmojiGrid tools in Experiments I, II, and III.

	Valence	Arousal
VAS_I–VAS_II	0.971 [0.921–0.986]	-0.076 [-0.312–0.175]
EmojiGrid_I–EmojiGrid_II	0.996 [0.994–0.998]	0.954 [0.923–0.972]
EmojiGrid_II–EmojiGrid_III	0.998 [0.979–0.994]	0.952 [0.911–0.973]
EmojiGrid_I–EmojiGrid_III	0.986 [0.975–0.992]	0.945 [0.903–0.968]


As noted before, Figure 6 shows a U-shaped relation between the mean arousal ratings obtained with both rating methods. A least-squares fit showed that the data points are indeed closely approximated by a quadratic function (red line in Figure 6; adjusted *R*-squared = 0.65). This surprising U-shaped relation suggests that the mean arousal measures resulting from both self-assessment tools may be linearly related if we neglect the polarity (pleasant vs. unpleasant) of the associated valence ratings. To test this hypothesis, we first distributed the arousal measures in two categories based on their corresponding valence values: one category associated with valence values below 50 (images rated as “unpleasant”) and one category associated with valence values above 50 (images rated as “pleasant”). The arousal ratings obtained with both methods showed a strong negative association (*r* = -0.73, *p* < 0.001) for images rated as unpleasant and a strong positive association for images rated as pleasant (*r* = 0.78, *p* < 0.001). Least-squares linear fits to the left or negative-valenced branch (adjusted *R*-squared = 0.52) and the right or positive-valenced branch (adjusted *R*-squared = 0.60) of the U-shaped relation between the arousal ratings obtained with both methods showed that the slopes of the negative (-1.1) and positive (0.9) valenced parts of the arousal curve had comparable absolute values. We computed an overall least-squares linear fit to the arousal data points (black line in Figure 6; adjusted *R*-squared = 0.76) after inverting (subtraction from 100) the arousal values corresponding to images with a valence that was rated below neutral (50). The resulting arousal ratings showed a strong overall positive association between both methods (*r* = 0.87, *p* < 0.001).

#### Valence Versus Arousal

**FIGURE 7:**
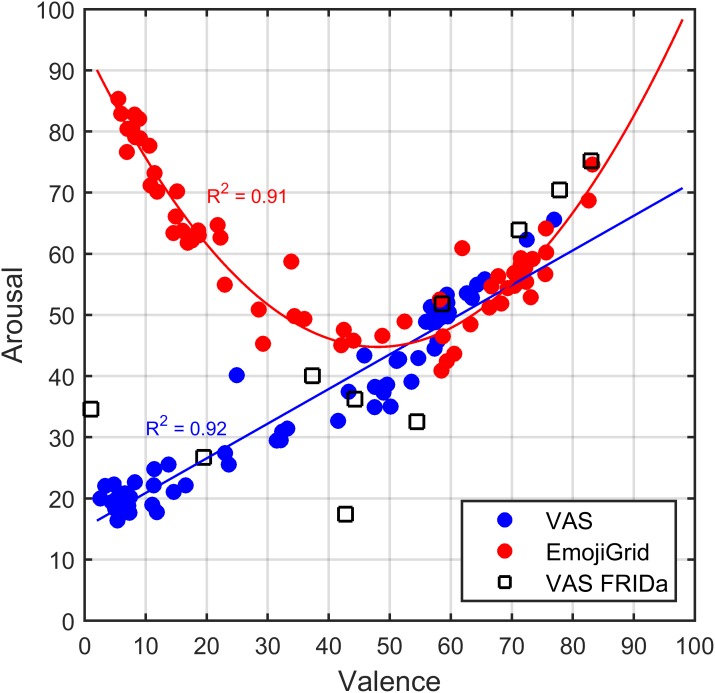
Relation between mean valence and arousal ratings for both measurement methods investigated in Experiment I. Blue dots: mean ratings obtained with VAS scales. Red dots: mean ratings obtained with the EmojiGrid. Black squares: mean ratings obtained with VAS scales from the study by Foroni et al. (2013). The blue and red lines represent a linear and quadratic fit to the VAS and EmojiGrid data points, respectively. The adjusted *R*-squared values represent the agreement between the data and the fits.

The results of Experiment I suggest that the participants interpreted both self-report assessment tools differently for unpleasant images. To follow up on this finding, we plotted the relation between the mean valence and arousal ratings obtained with both self-assessment tools in Figure 7. This figure shows the well-known U-shaped relation between valence and arousal for ratings obtained with the EmojiGrid. A least-squares fit showed that a quadratic function closely fits these data points (adjusted *R*-squared = 0.91). In contrast, the VAS tool appears to yield a linear relation between valence and arousal ratings. The valence and arousal ratings obtained with the VAS show a strong overall positive association (*r* = 0.96, *p* < 0.001). Figure 7 also shows the result of a linear least-squares fit to these data points (adjusted *R*-squared = 0.92).

To check whether the surprising linear relation between valence and arousal for the VAS self-assessment tool is an artifact of the present experimental procedure we compared our results with those of Foroni et al. (2013) who used the same VAS procedure to measure the valence and arousal for the 10 FRIDa images included in the present study (represented by the black squares in Figure 7). Figure 7 shows that the corresponding measurements for these 10 images are distributed along the linear least-squares fit to the data points obtained with the VAS tool. To verify this observation, we computed a linear correlation coefficient between the VAS ratings obtained in both studies (i.e., the present study and that of Foroni et al., 2013) for valence and arousal separately. Both the valence ratings (*r* = 0.87, *p* < 0.001) and the arousal ratings (*r* = 0.76, *p* < 0.001) obtained with the VAS tool showed a strong overall positive association between both studies. Hence it appears that the results of the VAS tool agree between both studies.

**FIGURE 8:**
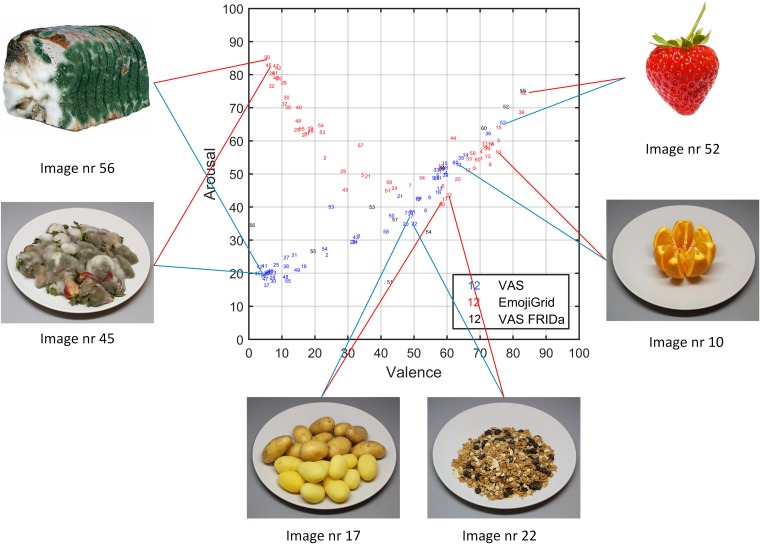
As Figure 7, where the dot symbols have been replaced by the stimuli indices (i.e., blue numbers represent mean ratings obtained with VAS scales, red numbers correspond to mean ratings obtained with the EmojiGrid, and black numbers represent mean ratings obtained with VAS scales from the study by Foroni et al., 2013). The VAS and EmojiGrid ratings for appetitive (nrs. 10, 52) and neutral (nrs 17, 22) stimuli are similar, while rating for unappetitive stimuli (nrs. 45, 56) are largely different (Images 52 and 56 reproduced with the permission of the copyright holder: Foroni et al., 2013).

To evaluate the face validity of the valence and arousal ratings we probed which items received extreme (the highest or lowest) and neutral valence and arousal ratings (some examples are shown in Figure 8). As expected, both methods yield the highest mean valence ratings for images of fresh fruit, chocolates, and pastries, while neutral ratings are obtained for images of raw onions, boiled eggs, and potatoes, and the lowest mean valence ratings correspond to images of rotten, molded, or contaminated food. However, the arousal ratings obtained with both methods only agree for neutral and positively valenced images, but not for negatively valenced images. Images of rotten, molded, or contaminated food yield the highest arousal ratings with the EmojiGrid, but the lowest ratings with the VAS arousal scale.

### Discussion

In this experiment, we found a close agreement for the valence ratings provided by the EmojiGrid and VAS tools. The ratings for arousal provided by both methods only agree for pleasant food items, but not for unpleasant ones.

Although the relation between subjective valence and arousal ratings depends both on personality and culture at the idiographic level (i.e., within individuals; Kuppens et al., 2017), the shape of this relation is typically characterized by a U-shape at the nomothetic or group level (i.e., both across persons and across a wide range of different stimuli such as sounds, music, paintings, images, movies, words, facial expressions, odors; Kuppens et al., 2013; Mattek et al., 2017). The results obtained with the EmojiGrid do indeed reflect this universal U-shaped relation between the valence and arousal ratings, in the sense that the arousal values monotonously increase from the center of the valence scale toward its extremes. Also, arousal values below neutral are scarcely reported, meaning that most food items are typically perceived as stimulating rather than de-activating. However, the VAS tool yields a linear relation between valence and arousal ratings, such that arousal monotonously increases with valence across the entire valence scale. This leads to the surprising result that food items with the lowest perceived valance are rated as least arousing. Our current finding agrees with previous (hitherto unexplained) findings that normative affective picture ratings obtained with labeled continuous VAS slider scales show a linear relation between valence and arousal (Foroni et al., 2013; Blechert et al., 2014), whereas ratings obtained with a SAM scale show a U-shaped relation (Marchewka et al., 2014; Riegel et al., 2017).

In this experiment, participants rated their feelings toward displayed food items on the affective dimensions of valence and arousal using two different tools (the VAS and EmojiGrid). For both tools, the meaning of the valence dimension was probably evident and directly related to the perceived pleasantness of the displayed products: the VAS tool clearly asked participants to rate the perceived positivity/negativity, and the facial expressions of the emoji in the EmojiGrid clearly displayed (dis-)pleasure. However, the understanding of the meaning of the arousal scale may have differed between both experimental conditions. Since the intensity of the facial expressions clearly increases in the upward direction along the vertical (arousal) axis of the EmojiGrid for each position along its horizontal (valence) axis, the participants probably correctly interpreted this dimension as the intensity of the associated stimulus valence. However, in the VAS condition, the participants may not have understood the meaning of the arousal concept in a food-related context. It has indeed previously been reported that people experience problems understanding the concept of arousal in the context of affective appraisal of food images (Ribeiro et al., 2005). In the current study, such a lack of understanding may have stimulated participants to copy their valence rating when responding their arousal rating. The spatial layout of the VAS may also have promoted such an answering bias: the two scales were presented one above the other, with the valence scale on top. Thus, the arousal response was always given after the valence rating and required a downward mouse movement. If participants were not sure about the meaning of “arousal” this layout made it even more attractive to minimize mouse movements and just click at the same horizontal position on both scales.

## Experiment II: Food Taste and Intensity Measured With Emojigrid and VAS

In Experiment I, we found that the EmojiGrid and VAS tools closely agree for the ratings of subjective valence. However, the ratings of subjective arousal only agreed for positively valenced (pleasant) images, but not for negatively valenced (unpleasant) ones. We hypothesized that this disagreement might reflect a lack of understanding of the meaning of the arousal concept.

The instructions used in Experiment I were image-focused and not internal state-focused: we asked for the affective qualities of the food items (how negative/positive and how arousing they were) but not for their immediate impact on the core affective state of the participants. It is however known that the affective qualities of stimuli are differently processed depending on whether they are relevant to the self or not (Schmitz and Johnson, 2007; Walla et al., 2013; see also Scherer, 2005).The appraisal of stimuli with self-relevance stimulates participants to assess their core affective state after engaging in a situated conceptualization based on stored representations of prior experiences (i.e., imagining an experience based on memories and knowledge; Lindquist et al., 2012). As a result self-relevance typically enhances the intensity and variation of subjective affective responses (Walla et al., 2013). In this experiment, we attempt to enhance the self-relevance of the task by asking participants to rate (i.e., imagine) the expected taste of the stimuli. We hypothesize that this will enhance the perceived arousal for negative valenced stimuli.

In the context of chemosensory (odor, taste, flavor) stimuli, valence typically measures the hedonic dimension (pleasantness), while self-rated arousal (the subjectively perceived internal state of activation or deactivation engendered by a stimulus) is strongly correlated with the perceived stimulus intensity (Bensafi et al., 2002; Winston et al., 2005; Wang et al., 2016). Perceived pleasantness and intensity are mediated by different brain mechanisms (Anderson et al., 2003; Small et al., 2003; Cunningham et al., 2004; Grabenhorst and Rolls, 2008). The orbitofrontal cortex evaluates the pleasantness of taste stimuli while the insular taste cortex processes the intensity and identity of the stimulus (Grabenhorst and Rolls, 2008; Grabenhorst et al., 2008). As a result, high level cognitive inputs (de Araujo et al., 2005; Grabenhorst et al., 2008) and selective attention to the affective or physical properties of a stimulus (Veldhuizen et al., 2007; Grabenhorst and Rolls, 2008) differentially modulate the subjectively perceived pleasantness and intensity of taste stimuli. The way instructions are formulated may therefore well affect the resulting ratings. Studies on the chemical senses typically adopt perceived intensity as a proxy for arousal (e.g., Small et al., 2003; Cunningham et al., 2004; de Araujo et al., 2005; Grabenhorst and Rolls, 2008; Grabenhorst et al., 2008; Rolls and Grabenhorst, 2008; He et al., 2016a). In this experiment we will follow this convention and we ask participants to rate not only the perceived valence (pleasantness) but also the perceived intensity of the expected taste of the stimuli. Note that this contrasts with the prevailing definition in emotion theory, where intensity is defined as the length (Euclidian norm) of the vector (with components along the two orthogonal circumplex dimensions valence and arousal) representing a given emotional state (Reisenzein, 1994).

In this experiment, we attempted to simultaneously clarify the meaning of the arousal concept and enhance the arousal response to negatively valenced stimuli in the VAS condition by asking participants to report the expected (imagined) valence and intensity of the taste associated with the perceived food item. We used exactly the same instructions in both (VAS and EmojiGrid) conditions. We hypothesized that this adjusted procedure would lead to a closer agreement between the subjective valence and arousal ratings obtained with both methods.

### Materials and Methods

#### VAS

**FIGURE 9:**
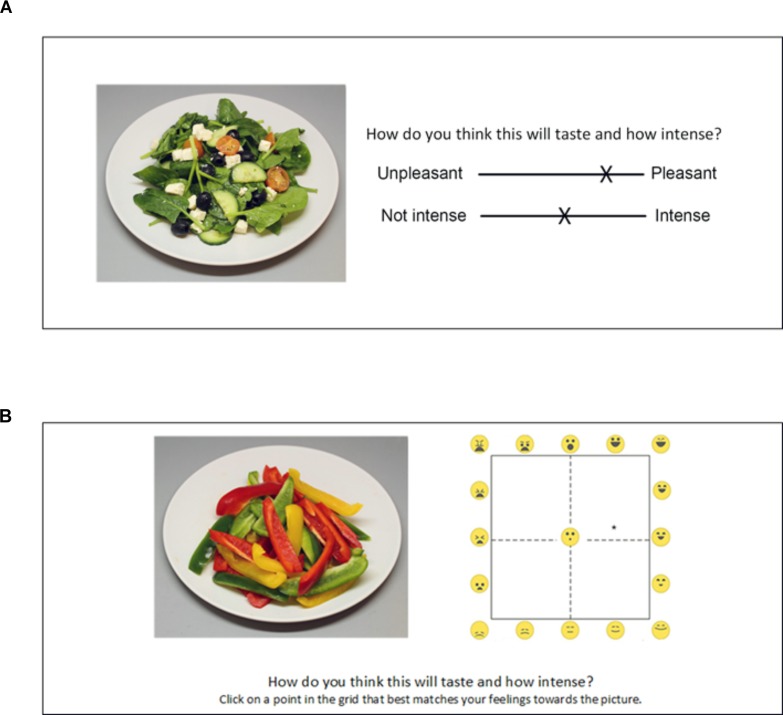
Screenshot of the VAS **(A)** and EmojiGrid **(B)** rating conditions in Experiment II.

In the VAS condition, the participants were asked to rate how each image made them feel by responding to the question “*How do you think this will taste and how intense?*” using two scales (Figure 9A): one for valence and the other for arousal. The extremes of the valence scale were labeled “*Unpleasant*” and “*Pleasant*” and the extremes of the arousal scale were labeled “*Not intense*” and “*Intense.*”

#### The EmojiGrid

In the EmojiGrid condition (Figure 9B), participants rated their affective feelings toward each image on the dimensions of valence and arousal by responding to the same question “*How do you think this will taste and how intense?*” using the EmojiGrid, with the additional instruction “*Click on a point in the grid that best matches your feelings towards the picture.*”

#### Participants

The total sample consisted of *N* = 117 participants, 45 males, and 72 females, with a mean age of *M* = 32.73 (*SD* = 15.72).

The sample in the VAS condition consisted of *N* = 58 participants, 29 males and 29 females with a mean age of *M* = 32.76 (*SD* = 15.06).

The sample in the EmojiGrid condition consisted of *N* = 59 participants, 16 males and 43 females with a mean age of *M* = 32.69 (*SD* = 16.47).

### Results

For each stimulus and for both self-assessment tools (EmojiGrid and VAS) we computed the mean response across all participants.

#### Effect of Wording (Experiment I Versus II)

To investigate whether the wording of the questions associated with both self-report tools affected the ratings, we separately computed the linear correlation between the valence and arousal ratings obtained in Experiments I and II. For the EmojiGrid, both mean valence (*r* = 0.98, *p* < 0.001) and arousal (*r* = 0.95, *p* < 0.001) ratings showed a strong overall positive association between both experiments, indicating that the wording of the questions had little or no effect on the subjective ratings obtained with this tool. For the VAS, the mean valence (*r* = 0.98, *p* < 0.001) ratings also showed a strong overall positive association between both experiments, but the arousal ratings showed no agreement (*r* = -0.1, *p* = 0.4).

To further quantify the effect of the wording used for the associated questions on both rating tools we also computed ICC estimates for the mean valence and arousal ratings obtained with the VAS and EmojiGrid used in Experiments I and II. The results (listed in Table 1) show that the valence and arousal ratings obtained with the EmojiGrid are independent of the actual wording used for the associated questions. The same holds for the valence ratings obtained with the VAS. However, there is no agreement between the arousal ratings between both experiments. Hence, it appears that the actual wording used its associated question strongly affects the outcome of the VAS arousal scale.

### EmojiGrid Versus VAS

**FIGURE 10:**
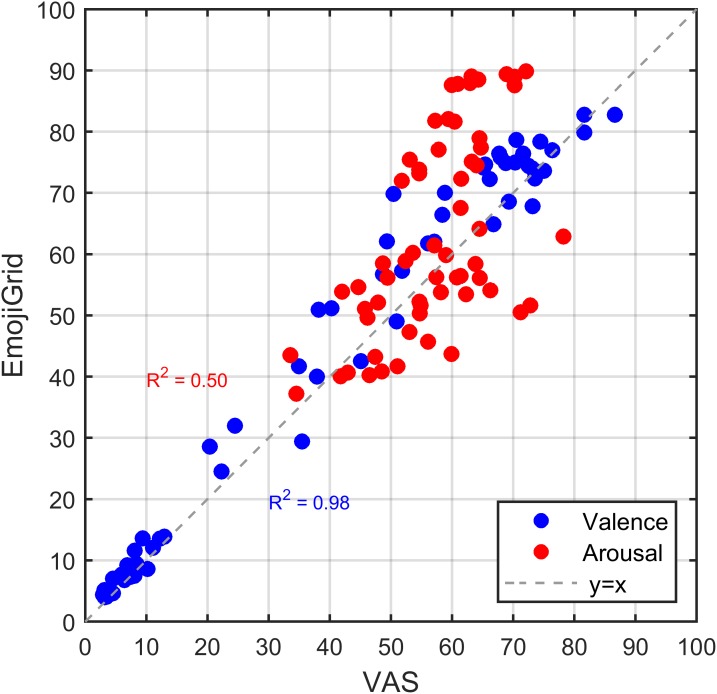
Relation between corresponding mean VAS and EmojiGrid ratings in Experiment II. Blue dots: mean valence ratings. Red dots: mean arousal ratings. The broken gray line with slope 1 represents full agreement between both methods. The adjusted *R*-squared values represent the agreement between the data and a linear fit with slope 1.

Figure 10 shows the relation between the mean valence and arousal scores obtained with both the VAS and the EmojiGrid in Experiment II. This figure clearly shows an overall linear relation between the ratings obtained with both methods, both for valence and now also for arousal. To illustrate this finding we computed a linear fit with slope 1, which yielded adjusted *R*-squared values of, respectively, 0.98 and 0.50 (Figure 10).

To further quantify the agreement between both methods we also computed ICC estimates and their 95% confidence intervals. The results (listed in Table 1) show that the valence ratings obtained with both (EmojiGrid and VAS) methods are in excellent agreement, while there is a good agreement between the arousal ratings.

#### Valence Versus Arousal

**FIGURE 11:**
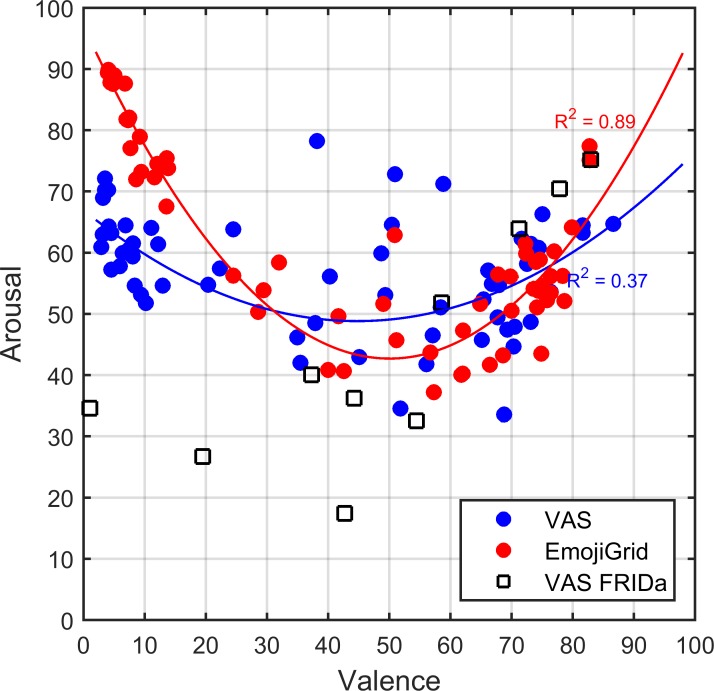
Relation between mean valence and arousal ratings for both measurement methods investigated in Experiment II. Blue dots: ratings obtained with VAS scales. Red dots: ratings obtained with the EmojiGrid. Black squares: ratings obtained with VAS scales from the study by Foroni et al. (2013). The blue and red lines represent quadratic fits to the VAS and EmojiGrid data points, respectively. The adjusted *R*-squared values represent the agreement between the data and the quadratic fits.

Figure 11 shows the relation between the mean valence and arousal ratings obtained in Experiment II with both self-assessment tools. This time we find the well-known U-shaped relation between valence and arousal measurements (Kuppens et al., 2013; Mattek et al., 2017), both for the VAS and the EmojiGrid tools. Least-squares fits to the valence and arousal ratings obtained with both methods show a strong quadratic relation for the EmojiGrid (adjusted *R*-squared = 0.89) and a significant quadratic relation for the adapted VAS tool (adjusted *R*-squared = 0.37). For comparison, we also plotted the VAS results for the 10 FRIDa images from the study of Foroni et al. (2013) in Figure 11. It is evident from these results that the adjusted VAS tool used in this experiment attributes significantly higher arousal values to images that are rated as unpleasant compared to the VAS tool used in Experiment I and in the study of Foroni et al. (2013).

**FIGURE 12:**
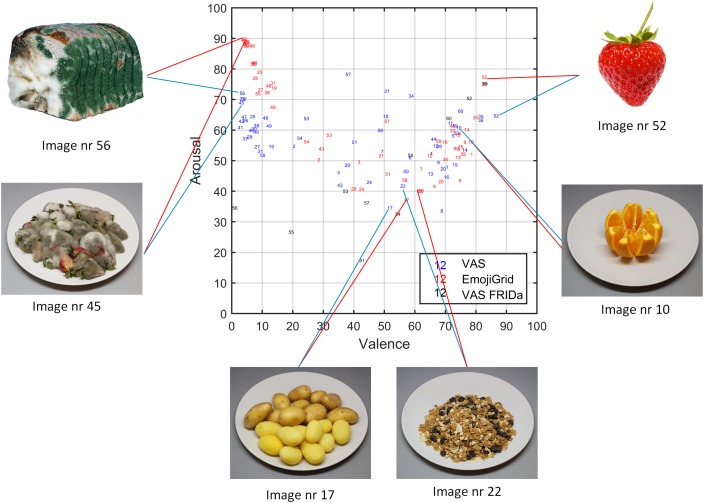
As Figure 11, where the dot symbols have been replaced by the stimuli indices (i.e., blue numbers represent ratings obtained with VAS scales, red numbers correspond to ratings obtained with the EmojiGrid, and black numbers represent ratings obtained with VAS scales from the study by Foroni et al., 2013). The VAS and EmojiGrid ratings are now similar for appetitive (nrs. 10, 52), neutral (nrs 17, 22), and unappetitive (nrs. 45, 56) stimuli (Images 52 and 56 are reproduced with the permission of the copyright holder: Foroni et al., 2013).

To evaluate the face validity of the valence and arousal ratings we again probed which items received extreme (the highest or lowest) and neutral valence and arousal ratings (some examples are shown in Figure 12). This time, both methods yield expected and similar results for both valence and arousal: the highest mean ratings are obtained for images of fresh fruit, chocolates, and pastries, while neutral ratings are obtained for images of raw onions, boiled eggs, and potatoes, and the lowest ratings correspond to images of rotten, molded, or contaminated food.

### Discussion

In this experiment, we attempted to clarify the meaning of the arousal concept by asking participants to rate the expected intensity of the taste associated with the perceived food item. In addition, we tried to enhance the self-relevance of the task by asking participants to rate (i.e., imagine) the expected taste of the stimuli. We hypothesized that these measures would serve to enhance the perceived arousal for negative valenced stimuli. We found that these procedural adjustments (1) indeed raised the mean perceive arousal levels of negatively valenced stimuli and (2) resulted in a closer agreement between the subjective valence and arousal ratings obtained with both the VAS and the EmojiGrid tools: both tools now yielded a U-shaped overall relation between the mean valence and arousal curves. Ratings obtained with the VAS arousal scale strongly depended on the actual wording used for its associated question. In contrast, the ratings obtained with the EmojiGrid were not affected by the framing of the associated question. This suggests that the EmojiGrid may be largely self-explaining and intuitive.

## Experiment III: Affective Food Response Measured With Emojigrid

To test the hypothesis that the EmojiGrid may be largely self-explaining and intuitive participants rated food pictures online using the EmojiGrid after minimal practice and without any further instructions, and we compared the results with those obtained in Experiment I (where participants were explicitly asked to rate the perceived valence and arousal of the food items) and Experiment II (where participants were explicitly instructed to rate the imagined taste valence and intensity of the food pictures).

### Materials and Methods

#### Procedure

**FIGURE 13:**
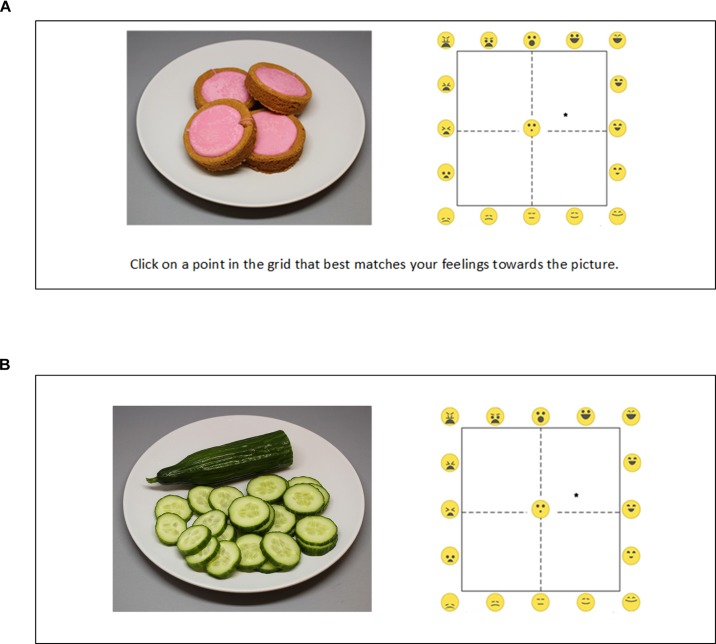
Screenshot of a practice trial **(A)** and an actual trial **(B)** in Experiment III.

In this experiment participants simply responded their affective feelings toward each image by clicking on an EmojiGrid that was presented without any further verbal instructions (Figure 13A). Before starting the actual experiment, they first performed two practice trials to familiarize them with the use of the EmojiGrid. The EmojiGrid in the practice trials was accompanied by the verbal instruction: “*Click on a point in the grid that best matches your feelings toward the picture*” (Figure 13A). This instruction was not shown in the actual experiment (Figure 13B).

#### Participants

The total sample consisted of *N* = 62 participants, 38 males, and 24 females, with a mean age of *M* = 27.16 (*SD* = 14.32).

### Results

**FIGURE 14:**
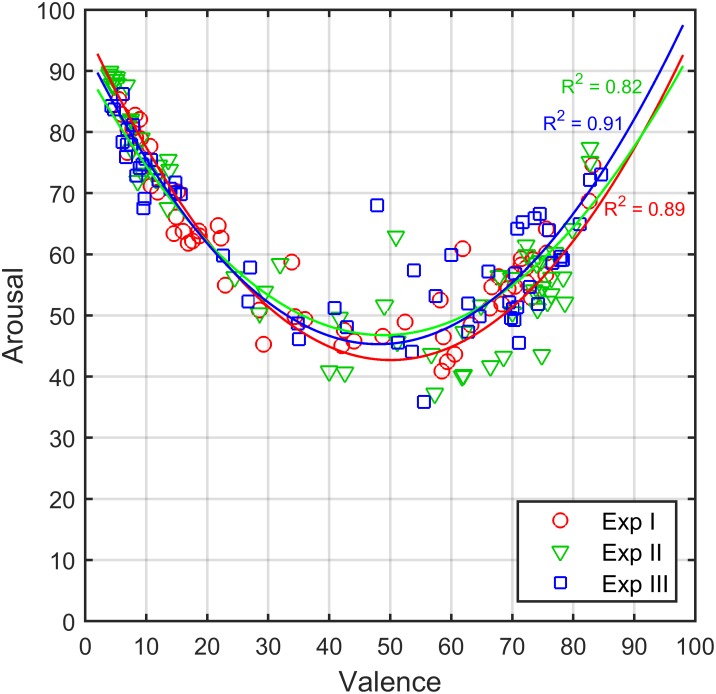
Relation between mean valence and arousal ratings obtained with the EmojiGrid in Experiments I, II, and III. The curved lines represent quadratic fits to the data points. The adjusted *R*-squared values represent the agreement between the data and the quadratic fits.

Figure 14 shows the relation between the mean valence and arousal ratings obtained with the EmojiGrid in this experiment, together with the previous results from Experiments I and II. This figure shows that the results for all three conditions closely agree. To quantify this agreement, we computed ICC estimates for the mean valence and arousal ratings obtained in the three different experimental conditions. The results (listed in Table 1) show that the valence and arousal ratings provided by the EmojiGrid are in excellent agreement between the different experimental conditions, independent of the presence or the wording of the instructions. This result agrees with the observation of Ares and Jaeger (2017) who found that question wording had little or no effect on affective food evaluation with emoji-based questionnaires.

### Discussion

The results from this experiment confirm our hypothesis that the EmojiGrid is largely self-explaining. Given the excellent agreement between the results of the first two experiments (EmojiGrid with instructions) and the third experiment (EmojiGrid without any explanation) it appears that users correctly interpret the valence and arousal dimensions, even without any explanation. It appears that the EmojiGrid is an intuitive instrument that requires no additional associated instructions (referring to either valence and arousal or taste and intensity) apart from the initial instructions to click on a point in the grid that corresponds to the user’s current feeling.

## General Discussion and Conclusion

How we feel about food determines to a large extent what, when, and how much we eat. Food evaluation studies therefore typically measure the principal affective dimensions of valence and arousal (e.g., Gil et al., 2009; Esteves et al., 2010; Swan et al., 2013; Miccoli et al., 2014; Piqueras-Fiszman et al., 2014; Hebert et al., 2015; den Uijl et al., 2016a; Wang et al., 2016; Woodward et al., 2017). Measures of food-evoked emotions are therefore an essential and valuable source of information for product development and marketing. Hence there is a need for an efficient food-specific self-report tools that produce reliable and valid data. In this paper, we introduced the EmojiGrid as a promising new efficient graphical self-report tool to measure food-related affective associations. The EmojiGrid is a Cartesian grid that is labeled with emoji showing food-related facial expressions. Users can report their subjective ratings of valence and arousal by marking the location on the grid that corresponds to the emoji (facial expression) that best represents their affective state after perceiving a given food or beverage. The tool is both intuitive (the facial expressions speak for themselves and don’t need additional labels) and efficient (the two affective dimensions are measured with a single response).

In this study we performed three experiments to validate the EmojiGrid as a self-report tool for measuring food-evoked affective feelings. In summary, the aims and key findings of these three experiments are as follows. In two comparative evaluation studies, we first compared the performance of the EmojiGrid with conventional VAS. The results of the first experiment showed that the valence ratings provided by the EmojiGrid closely agree with those provided by a standard VAS tool, whereas the arousal ratings provided by both methods only agreed for pleasant food items but not for unpleasant ones. Unlike the EmojiGrid, the VAS ratings did not show the universal U-shaped relation between the mean valence and arousal ratings at the group level that is typically reported in the literature. We hypothesized that this disagreement probably resulted from a lack of the participants’ understanding of the arousal concept. In a follow-up experiment, we attempted to clarify the meaning of the arousal concept and to enhance its self-relevance by asking for the expected intensity of the taste associated with the perceived food item. After this adjustment, the valence and arousal ratings obtained with both tools (VAS and EmojiGrid) agreed more closely and both showed the universal U-shaped relation between the valence and arousal. In a final (third) experiment we established that the EmojiGrid yields valence and arousal ratings that do not depend on the actual wording or presence of further instructions. This result contrasts with the finding that ratings obtained with VAS arousal scales strongly depend on the exact formulation of the associated question.

Cross-cultural studies on food-related emotions are becoming increasingly important as a result of the globalization of food products (Meiselman, 2013). However, verbal self-assessment tools typically pose difficulties for cross-cultural research since emotion words are often not directly equivalent in different languages (Wierzbicka, 1999). In addition, consumers from different cultures tend to use emotions terms differently (van Zyl and Meiselman, 2015, 2016). The non-verbal and intuitive EmojiGrid may be a valuable tool for cross-cultural studies since it is independent of language and requires only minimal initial instructions (“*Click on the grid*”), exploiting the fact that facial expressions of emotions (e.g., joy, disgust) are largely universal.

Jaeger et al. (2018c) recently found that the use and interpretation of emoji is not influenced by age or frequency of emoji use, suggesting that the EmojiGrid may be a useful tool for users of all ages.

## Limitations of This Study

The emoji used for the EmojiGrid in this study all had the same size, shape, and color. Only their facial (mouth and eyes) features were varied systematically and in a straightforward (simple) way to create various general emotional expressions. It may be possible to design emoji (possibly more elaborated and created by cartoon artists) that are more food-related. Future studies should investigate the effects of graphical emoji properties like size, shape, and color on the interpretation of their facial expressions and ultimately on the resulting affective ratings.

The neutral emoji label in the middle of the grid may have had a repulsive effect on the observer response (people may hesitate to click on a face), thus causing a greater variation in the data for (near) neutral stimuli. Future experiments could investigate whether a neutral midpoint is essential.

## Future Research

This study suggests that the EmojiGrid can indeed capture the affective dimensions of an emotional response to food. Whether such a measure does indeed enable a better prediction of food choice than a unidimensional hedonic rating should be the topic of future studies.

An obvious extension to the present research will be to use the EmojiGrid in food evaluation studies in cross-cultural studies. This involves the investigation of cultural influences on the interpretation of emoji meaning. Emoji may in principle elicit a more intuitive and affective response, which may be particularly useful when testing Asian populations who may be culturally biased to avoid negative scale anchors (Chen et al., 1995; Lee et al., 2002, see also Jaeger et al., 2018a).

Children are an important consumer group with special needs. Currently there is no tool for the assessment of children’s emotional associations with food (Gallo et al., 2017). Emoji provide a visual display of emotion, making them in principle also a useful tool for populations such as children who do not have the vocabulary to express their emotions. Initial studies have indeed shown that children are quite capable and like to use emoji to characterize their emotions in relation to food (Gallo et al., 2017).

The EmojiGrid may also be a useful tool to evaluate other affective stimuli such as photographs, paintings, music, smells, and tactile signals, etc. In consumer research, the EmojiGrid can also be used to assess the emotional response to for instance oral care products (Chen et al., 2018), fragrances (Churchill and Behan, 2010), fabrics (Wu et al., 2011), affective ambiences or servicescapes (Kuijsters et al., 2015), etc.

Similar to the AffectButton (Broekens and Brinkman, 2013) and EMuJoy (Nagel et al., 2007) the EmojiGrid may enable users to continuously report perceived affect in human–computer interaction studies by moving a mouse controlled cursor over the support of the grid. While these existing tools require the user to successively explore the entire affective space to find the desired expression each time a response is given, the EmojiGrid provides an instantaneous overview of the affective input space. This feature may be useful for the affective annotation of multimedia (Runge et al., 2016) or personalized affective video retrieval (Xu et al., 2008; Lopatovska and Arapakis, 2011), for real-time affective evaluation of entertainment (Fleureau et al., 2012) or as an affective input tool for serious gaming applications (Anolli et al., 2010).

## Author Contributions

AT and VK had the original idea for this study. AT and IdK designed the EmojiGrid. AT, DK, SU, and IdK designed the experiments. SU programmed the online experiments. DK, IdK, and SH collected the data. AT, DK, IdK, and SH analyzed the data. AT produced the figures and wrote the draft manuscript. AT, DK, A-MB, VK, and JvE critically revised the manuscript on article structure, logical organization, and language.

## Conflict of Interest Statement

The authors declare that the research was conducted in the absence of any commercial or financial relationships that could be construed as a potential conflict of interest.

## Footnotes

1 http://www.mathworks.com

## Supplementary Material

The EmojiGrid, the 60 food images used as stimuli in the experiments, full documentation of the experimental procedures, and an Excel file with the results are all available from the Figshare Repository (https://figshare.com/articles/_/6463151) with doi: 10.6084/m9.figshare.6463151.
